# Incorporating date palm fibers for sustainable friction composites in vehicle brakes

**DOI:** 10.1038/s41598-024-73275-1

**Published:** 2024-10-05

**Authors:** Zeina Ammar, Mahmoud Adly, Somia Yassin Hussain Abdalakrim, Sherif Mehanny

**Affiliations:** 1 Mechanical Design & Production, Faculty of Engineering, Cairo University, Cairo, 12613 Egypt; 2 The Key Laboratory of Advanced Textile Materials and Manufacturing Technology of Ministry of Education, Zhejiang Sci-Tech University, Xiasha Higher Education Park Avenue 2 No. 928, 310018 Hangzhou, China

**Keywords:** Bio-composites, Brake pads, Palm date, Natural fiber, Tribological properties, Mechanical engineering, Biomaterials

## Abstract

The demand for eco-friendly materials in automotive components has spurred research into natural fibers as sustainable alternatives for brake pads. This study examines the potential of date palm fibers, particularly the palm frond midrib (PFM), in brake pad composites. The effects of epoxy, PFM, and calcium carbonate on the composites’ mechanical and tribological properties were analyzed. The optimal formulation (25% epoxy, 30% PFM, 35% calcium carbonate) exhibited superior properties, including a hardness of 87 HRB, wear rate of 1.5E-03 mg/mm, and COF of 0.73, surpassing commercial pads. Additionally, an inverse relationship between PFM/calcium carbonate content and compressibility was observed, with increased calcium carbonate enhancing wear resistance. This research underscores the potential of utilizing date palm resources in eco-friendly brake manufacturing, reducing the environmental and health impacts of traditional materials.

## Introduction

The braking system is a crucial safety component of automobiles, designed to decelerate or halt the vehicle. Central to this system, brake pads convert kinetic energy into thermal energy via friction. Consequently, the selection of friction materials in brake pads is pivotal for achieving optimal braking performance and safety^1,2^. Composites have historically emerged as a versatile class of materials, providing tailored properties for demanding applications^3^. Friction composite materials used in brake pads typically comprise four main components: binder, fillers, friction modifiers, and fibers^4^. Fibers are particularly significant as they enhance strength, influence fade resistance, and improve braking efficiency^2,4^. Several types of friction materials are employed in brake linings, including metallic, carbon-carbon composites, and organic polymeric composites^2^, with the latter being predominant in light-duty automotive applications. Traditionally, asbestos fibers were extensively utilized in organic brake pads due to their advantageous properties^5^. However, the carcinogenic nature of asbestos necessitated the development of safer alternatives^6^.

The environmental and health risks of asbestos have spurred the search for alternative materials. However, many alternatives are energy-intensive or not fully developed, often merely shifting environmental impacts^7^. Consistent environmental assessments are needed to find solutions that minimize the environmental impact of asbestos-containing waste (ACW) throughout its lifecycle^8^.

Life cycle assessment (LCA) is crucial for evaluating the environmental impacts of processes or products across their entire lifecycle^9–11^. However, few LCA studies address ACW management due to the lack of methods for assessing asbestos emissions’ impact on soil, water, and air^12,13^. Recent advancements in asbestos-containing materials (ACMs) management include risk maps, encapsulation, and elimination of ACMs, and developing end-of-life strategies for ACW^8^. Proposed end-of-life scenarios include thermal inertization and hazardous waste landfill disposal, with findings integrated into the USEtox 2.0 impact assessment method^8^. Inertization is favored for its reliability and potential to repurpose inert material as a secondary raw material, reducing environmental harm^8^.

Natural fiber reinforced composites (NFRCs) offer greater sustainability compared to synthetic fiber composites and have significant potential to enhance the sustainability of composite materials^14^. Utilizing fruit residuals from processing industries as frictional materials for brake pads mitigates environmental issues and repurposes waste for economic benefits, reducing the detrimental impact of asbestos and greenhouse gas emissions from traditional composting methods^15–19^.

The shift towards sustainability has highlighted the potential of natural fibers, derived from renewable resources such as plants, as a promising alternative to conventional brake pad materials^20,21^. Natural fibers have emerged as a viable substitute for asbestos in brake pad materials^20,22,23^. They provide advantages including cost-effectiveness, low density, biodegradability, and environmental sustainability^24^. In addition to replacing asbestos, the use of natural fibers supports the broader movement towards sustainable materials that minimize waste and environmental impact^25^.

The utilization of natural fibers as reinforcement materials in composite structures has garnered significant attention due to their inherent sustainability and environmental benefits. However, their durability, particularly under the demanding conditions encountered in automotive applications, remains a critical concern. Recent studies have underscored the necessity of addressing this challenge to ensure the long-term viability of natural fiber composites in such environments.

For instance^26^, conducted a comprehensive investigation into the long-term performance of natural fiber-reinforced composites, focusing on their resistance to environmental degradation and mechanical wear. Additionally^27^, provided valuable insights into the mechanical behavior of these materials under various stress conditions, further emphasizing the need for durability enhancements. Collectively, these studies highlight the imperative of developing strategies to improve the durability of natural fiber composites, thereby enabling their wider adoption in demanding applications such as automotive brake pads.

Expanding the exploration of natural fibers in brake pads, it is noteworthy that their potential extends beyond this specific application. Numerous studies have documented the enhanced tribological (friction and wear) and mechanical properties of NFRC materials. These studies have investigated various natural fibers, such as palm kernels, banana peels, coconut (coir) fibers, bamboo fibers, wood powder, flax fibers, pineapple fibers, hemp fibers, and pine needle fibers as reinforcement components within NFRCs. Typically, phenolic resin or epoxy serves as the binder material in these composites^28^.

Resins, alongside fibers, are crucial components in composite materials. The composition of resins, such as epoxy and phenolic, plays a significant role in determining the properties and applications of the composites. Epoxy Resins are typically formed from the reaction of an epoxide with polyamine. They are known for their exceptional mechanical strength, strong adhesion, and high chemical resistance. Traditionally, epoxy resins are derived from petroleum-based sources, specifically from the reaction between epichlorohydrin and bisphenol A (BPA)^29^. However, there is a growing interest in bio-sourced epoxies, which are derived from renewable resources like cardanol (from cashew nutshell liquid) and lignin^30,31^. These bio-based epoxies offer similar mechanical properties to conventional epoxies while being more environmentally friendly.

Phenolic Resins are made from phenol and formaldehyde. They are valued for their high heat resistance, flame resistance, and good mechanical strength. Traditionally, phenolic resins are also derived from petrochemical sources. However, efforts are being made to utilize renewable materials in their production. Bio-sourced phenolic resins can be partially made from natural products such as cashew, tannin, starch, and lignin^32^. These bio-based alternatives help reduce dependence on petrochemical raw materials and contribute to sustainability. The potential for bio-sourced resins is significant as they offer a more sustainable and eco-friendly alternative to traditional petroleum-based resins. By incorporating renewable resources, the environmental impact of composite materials can be reduced, paving the way for greener and more sustainable industrial practices^33^.

This body of research underscores the diverse range of natural fibers that have been effectively utilized in the fabrication of NFRC materials, highlighting their potential to enhance both mechanical strength and tribological performance. These findings are crucial for the development of eco-friendly and high-performance composite materials in various applications, including automotive components and construction materials. The integration of natural fibers in composites aligns with the broader trend towards sustainable materials development, demonstrating the potential to reduce dependence on synthetic materials and mitigate environmental impact.

The examination of natural fibers in brake pads opens a comprehensive avenue for literature review, revealing significant advancements and the benefits of incorporating these fibers into friction composite materials. This review aims to explore the diverse types of natural fibers, their tribological and mechanical properties, and their potential to replace hazardous materials like asbestos. By understanding the current state of research and development in this field, we can better appreciate the innovation, safety, and sustainability these materials bring to modern engineering solutions. This review will delve into the latest studies, highlighting both the achievements and the ongoing challenges in utilizing natural fibers for friction materials, setting the stage for future research and applications.

The integration of palm kernel fibers (PKs) into friction composites has demonstrated that these materials can meet or exceed the standard requirements for commercial brake performance. Specific formulations of these composites have shown superior properties compared to others tested, indicating that palm kernel fibers are a viable replacement for asbestos in brake pad production. The binding ingredients in these composites include epoxy resin, aluminum oxide, carbon, and calcium carbonate. The fiber content in these composites varies from 15 to 40%, with the optimal fiber content determined to be 10% by weight. The manufacturing process involves cold pressing at 100 kN and 25 °C for 2 min^34^.

Experimental results indicated that wear rates for all samples increased with higher operating speeds, a common behavior attributed to increased contact pressure. Notably, certain PKs samples outperformed commercial brake pads in terms of wear resistance at high speeds. The coefficient of friction remained relatively stable across varying speeds for all brake pads, a stability attributed to the absence of steel fibers, which are known to affect the friction coefficient. Additional properties, including porosity, hardness, noise levels, moisture absorption, and specific gravity, all demonstrated behaviors within acceptable ranges for brake performance. The surface roughness of the PKs brake pads met or exceeded industry standards. These findings suggest that palm kernel fibers hold significant potential as a material for brake pad manufacturing, offering promising results in key performance areas^34^.

In another formulation, Type-1 composite consists of phenolic resin, carbon, aluminum oxide, and PKs, with the fibers treated by soaking in NaOH for 24 h before use. The optimal fiber content for this composite was found to be 50% by weight. Type-2 composite also includes a 24-hour NaOH treated fiber component and consists of phenolic resin, carbon, aluminum oxide, wheat, and Nile rose, with varying weight percentages for each ingredient^35^.

The study revealed several key findings. In terms of wear properties, Type-2 composites showed improvement with the addition of 5% wheat to a 30% volume fraction of fiber. Conversely, Type-1 composites achieved optimal wear properties by increasing the palm kernel volume fraction to 50%. Type-2 composites exhibited maximum hardness when 10% wheat and 15% Nile rose were added to 25% palm kernel fiber. This hardness level was comparable to that of Type-1 composites with 50% PKs. Additionally, Type-1 composites with 50% PKs demonstrated the lowest oil absorption after immersion for five days. In Type-2 composites, a combination of 2% Nile rose, 3% wheat, and 5% PKs led to a reduction in oil absorption^35^.

Alternative composite consists of PKs and phenolic resin, along with various ingredients including acrylic fiber, recycled wood fibers, steel fiber, calcium hydroxide, nitrile butadiene rubber, chloroprene rubber, calcium oxide, silicon carbide, artificial carbon, molybdenum disulfide, and barium sulfate. Prior to manufacturing, the PKs undergo sodium hydroxide soaking for 24 h. Researchers varied fiber content from 0 to 10% by weight, determining an optimal fiber content of 5% ^36^.

The study revealed several key findings about the properties of the developed composites. Density decreased as palm kernel fiber content increased. This is likely due to the higher density of the fibers and the introduction of space fillers, resulting in greater porosity. The enhanced porosity is attributed to the palm kernel fibers’ larger particle size and their uniform distribution within the matrix. Correspondingly, hardness decreased as palm fiber content increased, reflecting the more porous nature of the composites. Generally, compressibility increased, although minor deviations were observed^36^.

The study also conducted a Chase test analysis, specifically examining fade-recovery cycles. Results indicated that the coefficient of friction values increased up to 149 degrees Celsius. Beyond this temperature, different frictional trends emerged across composites with varying palm kernel fiber content. Overall, the research suggests that composites containing 5% palm kernel fiber exhibited superior frictional performance and reduced wear rates, making them a promising choice among the developed formulations^36^.

The incorporation of banana peels in friction composite brake pad as a sustainable replacement was also investigated. Alternative composite consists of includes carbon, calcium hydroxide, calcium carbonate, aluminum oxide, molybdenum disulfide, antimony sulfide, magnesium oxide, silicon carbide, steel wool, PAN fiber, and phenolic resin, with respective weight percentages. Fiber content variation ranges from 0 to 21% by weight, with the optimum fiber content identified at 7% ^37^. The manufacturing process is hot pressing, subjecting the composition to conditions of 15 MPa pressure, 150 °C temperature, for a duration of 10 min^37^.

The study examined the physical and mechanical properties of four brake pad samples, noting the highest density in sample of 21 fiber weight% and consistent hardness across all samples due to uniform pressure during compaction. While surface roughness met acceptable standards^37^.

Friction and wear tests revealed that sample of seven fiber weight% outperformed others, maintaining a stable, higher friction coefficient with minimal wear under demanding conditions. In contrast, samples of zero, 14, and 21 fiber weight% exhibited decreasing coefficients of friction due to insufficient binding between phenolic resin and banana fiber at elevated temperatures. The optimal reinforcement of banana fiber is seven weight% improved binding stability and friction coefficient^37^.

In summary, the study demonstrated that the inclusion of banana fiber, particularly in combination with phenolic resin at elevated temperatures, enhanced binding capabilities, resulting in improved fade resistance and a consistent friction coefficient. This suggests its suitability for use in brake pad materials^37^.

Further investigation for this research was done after some changes in the components and its weight% while. The composition comprises calcium carbonate and barium sulfate, PAN fiber, calcium silicate, steel fiber, carbon, molybdenum disulfide, aluminum oxide, silicon carbide, magnesium oxide, and phenolic resin, with respective weight percentages. Fiber content varies from 4 to 16% by weight, with the optimum fiber content identified at 12% ^38^.

The study conducted a comprehensive characterization of four brake pad samples, focusing on their physical and mechanical properties. The samples exhibited a density range of 2.26–2.32 g/cm³, with slight hardness increases as banana fiber content rose. In friction testing, the COF increased significantly with higher disc speeds, influenced by banana fiber and phenolic resin content. Sample of 12 fiber weight% displayed superior fade resistance and frictional performance. Interface temperatures increased with time and disc speed, following frictional heating principles^38^. The specific wear rate showed higher wear at 450–750 rpm but a slight decrease at 750–1050 rpm, attributed to material property changes and frictional heat during braking. This comprehensive characterization emphasizes the need to optimize composition for improved braking performance and reduced brake fade, without delving into thermal properties^38^.

Thus, key findings include the significant influence of banana fiber proportions on mechanical and tribological properties, with increased fiber content leading to higher hardness. Friction tests revealed improved performance, and brake pad samples denoted as sample of 12 fiber weight% exhibited superior performance and moderate wear rates when incorporating banana fiber with phenolic resin as a modified binder^38^.

The incorporation of coconut and bamboo fiber in friction composites demonstrated significant potential as a sustainable replacement for asbestos in brake pad manufacturing, with performance metrics meeting or exceeding those of commercial pads. Results obtained in various studies are presented in (Tables 1 and 2).

While existing research on natural fibers for brake pads offers valuable insights, a significant gap exists regarding date palm fibers. This presents a unique opportunity, especially in regions like the Middle East and North Africa (MENA) where date palms are abundantly cultivated^39^. MENA boasts a staggering 90% of the estimated 120 million date palm trees cultivated globally^39^, with Egypt itself contributing an impressive 12 million^40,41^. These trees are a treasure trove of lignocellulosic biomass, a renewable and eco-friendly material. However, the potential of Palm date fibers remains largely unexplored in brake pad applications. This presents a unique opportunity to investigate a locally sourced, sustainable material that could make a revolution in industry.

Palm Date fibers offer a sustainable and cost-effective reinforcement material. Each date palm tree yields approximately 40 kg of waste annually. This waste includes fibrous materials, dried fruits, and seeds. Unfortunately, a significant portion of this valuable biomass is burned on farms annually due to the lack of effective processing strategies^42^ .However, there are substantial opportunities for the sustainable utilization and valorization of palm waste.

Brake pads are crucial components in automotive safety, yet conventional materials often rely on non-renewable resources and exhibit suboptimal performance in certain conditions. This study investigates the use of Palm Frond Midrib (PFM) fibers, a renewable and readily available material, in brake pad composites. The research aims to determine whether PFM can enhance the mechanical and tribological properties of brake pads while providing an eco-friendly alternative to traditional materials.

**Table 1:** Illustrates coconut/coir and bamboo content variation, the optimum fiber content, manufacturing process of braking pads and post treatment.

Friction composite	Other ingredients	Fiber content variation	Optimum fiber content	Manufacturing process	Post treatment
Coconut fiber/ PR ^43–45^	[Al, C, ZrO_2_, TiO_2_, PR, C6H12N4] [(20, 15), 5, 2, 11, 40, 6%]	5, 10 Wt.%	10%	Cold pressing (20KN) followed by hot pressing (20 KN, 200 ° C, 1 h)	100 °C, 8 h
	[Al, C, ZrO_2_, TiO_2_, SiC, PR, C6H12N4] [(14, 9), 10, 2, 13, 10, 40, 6%]		–	10 N, 200 °C, 3 min	200 °C, 2.5 h
	[Al, C, ZrO_2_, SiC, TiO_2_, PR] [(25, 20, 15, 10), 10, 2, 10, 13, 40%].	0, 5, 10, 15 Wt.%	–	10 N, 200 °C	200 °C, 2.5 h
	[Al, C, ZrO_2_, SiC, Al_2_O_3_, PR] [(25, 20, 15, 10), 10, 2, 10, 13, 40%]	0, 5, 10, 15 Wt.%			
Coir fiber/ PR ^46,47^	[Al, SiC, C, Al_2_O_3_, ZrO_2_, Paper ash, PR] [(45, 39, 33, 2, 11), 20, 10, 13, 2, (0, 1, 2, 3, 4), 10%)	0, 5, 10, 15, 20 Wt.%	5%	200 N, 170 °C, 1 min	200 °C, 5 h
	[PR, SF, C, LF, Slag waste] [10, 10, 5, 10, (60, 55, 50, 45%)]	5, 10, 15, 20 Wt.%	–	Cold pressing (10 MPa) followed by hot pressing (15 MPa, 155 °C, 10 min)	170 °C, 4 h
Coconut fiber/ wood powder ^48,49^	[Wood powder, polyester resin] [(40, 30, 20, 10, 0),60%]	0, 10, 20, 30, 40 Wt.%	–	2 tons, 60 min	–
	[Wood powder, polyester resin, green mussel shell], [(30, 20, 10), 50, 10]	10, 20, 30 Wt.%	–	2 tons, 60 min	–
Coconut fiber/ ER^50,51^	[ER, Al_2_O_3_, MgO] [46, 28, 6]	20 Wt.%	–	24 Kgf.m, 180 °C, 40 min	–
	[ER, Mgo, Al_2_O_3_] [46, (11, 6, 1), (23, 28, 33%)]	20 Wt.%	S2	1000 Kgf.m, 200 °C, 20 min	–
Bamboo fiber/ ER^51,52^	[ER, MgO, Al_2_O_3_] [40, (11, 6, 1), (20, 25, 30%)]	29 Wt.%	S_2_	1000 Kgf.m, 200 °C, 20 min	
	[MgO, ER] [(30, 25, 20), (30, 25, 20%)]	40, 50, 60 Wt.%		200 psi(≈ 1.4 MPa), 8 min	180 °C, 30 min
Bamboo fiber/ PR^53^	MF, GF, PR, VP, FIP, BaSO_4_, PC, C, Al_2_O_3_, Sb_2_S_3_, FP, Zn(C_18_H_35_O_2_)_2_, CB.	0, 3, 6. 9,12 Wt.%	3 Wt.%	–	–

*PR* phenolic resin, * ER* epoxy resin, * SF* steel fiber, * LF* lapinus fiber, * MR* mineral fiber, * GF* glass fiber, * VP* vermiculate powder, * FIP* foam iron powder, * PC* petroleum coke, * FP* friction powder, * CB* carbon black, * Wt.%* weight%, * S* sample.

**Table 2:** Illustrates the effect of coconut/ coir and bamboo fiber on tribological and mechanical properties of braking pads.

Friction Composite	T-M/St	COF	WR	WR Unit	T-M/St	Hardness	T-M/St	*P*%	T-M/St	Compressive Strength/ Compressibility	TC/ TR
Coconut fiber/ PR ^43–45^	–	FWt↑ → COF ↑	FWt↑ → WR↓	g	–	FWt ↑ → HRS↑	–	FWt ↑→ P% ↓	–	–	–
	Pin on the disc	–	FWt↑ → WR↓	mm, g	–	–	–	FWt ↑→ P% ↓	–	–	–
	–	–	–	–	HRC B scale. E N ISO 6508-1	FWt ↑ → HRC↓ Except S_1_	–	–	–	–	–
	–	–	–	–		FWt ↑ → HRC↓ Except S_1_	–	–	–	–	
Coir fiber/ PR ^46,47^	Chase test	–	FWt↑ → WR↑	Wt. loss%	HRS S scale. MS 474	FWt ↑ → HRS↓ Except S_1_	Tensiometer JIS D4428	FWt ↑→ P% ↓ up to certain point then ↑	UTM	Almost the same	–
	Krauss machine	–	FWt↑ → WR↑	g, mm	HRR R scale. ASTM D785	FWt ↑, Slag waste↓→ HRR↓	JIS D4428	FWt ↑, Slag waste↓→ P%↑		FWt ↑, Slag waste↓→ Compressibility↑	–
Coconut fiber/ wood powder ^48,49^	Test rig	–	FWt↑ → WR↓	g	VHN ASTM E92	FWt ↑ → VHN↓	–	–	–	–	–
	–	–	–	–		FWt ↑ → VHN↓	–	–	–	–	–
Coconut fiber/ ER^50,51^	Pin on disk/ASTM D 1894–001, ASTM G 99-95a.	COF = 0.41 < commercial pad (0.43)	2.99 × 10^− 7^ > commercial pad (1.77 × 10^− 7^)	cm^3^/Nm	Shore D durometer. ASTM F1957-99	97.27 HD < commercial 99.05HD)	IS0 15,484	19.72% > commercial (11.89%)	–	–	
	Pin on disk/ASTM G 99-95a.	No trend	Mgo↓, Al_2_O_3_↑→ WR↑	mm^3^/N.mm	HRB ASTM E18-15	Mgo↓, Al_2_O_3_↑→HRB↓ Except S_1_	–	–	Stress simulation (FEA)	Mgo↓, Al_2_O_3_↑→σ ↑	Mgo↓, Al_2_O_3_↑→TR↓
Bamboo fiber/ ER^51,52^	Pin on Disk/ASTM G 99-95a	No trend	Mgo↓, Al_2_O_3_↑→ WR↑	–	–	Mgo↓, Al_2_O_3_↑→HRB↓ Except S_1_	–	–		Mgo↓, Al_2_O_3_↑→σ ↓	Mgo↓, Al_2_O_3_↑→TR ↑
	ASTM D3702-94	–	FWt↑ → WR↑	g/mm^2^.s	HRR ASTM D785-03	FWt ↑ →HRR↑	–	–	–	–	–
Bamboo fiber/ PR^53^	Speed friction tester	FWt↑ → COF↓	FWt↑ → WR↑	µm^3^/ (N-µm)	–	–	–	–	–	–	–

↑ increasing, ↓ decreasing, * FWt* fiber weight, * HRB* rockwell hardness B scale, * HRR* rockwell hardness R scale, * UTM* universal testing machine, * HRC* rockwell hardness, * VHN* vicker hardness, * HRB* rockwell hardness B scale, * HRR* rockwell hardness R scale, * HRS* rockwell hardness S, * HD* shore hardness scale D.

## Results

### Mechanical properties

#### Force vs. displacement

**Fig. 1:**
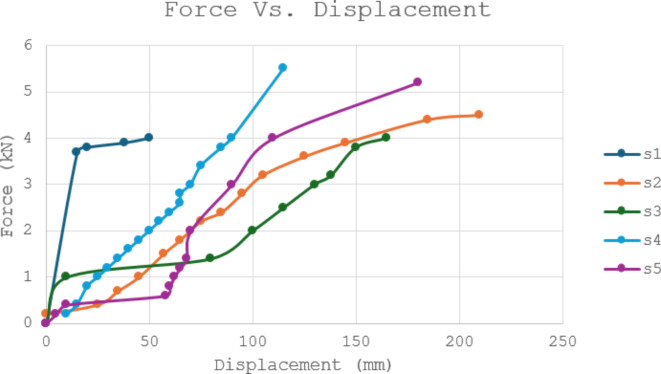
Force vs. displacement curves for different formulations of the new formulated brake pads.

To gain a deeper understanding of the material’s response to applied force, force vs. displacement curves were generated for each newly formulated brake pad (Fig. 1). These curves provide valuable insights into the material’s deformation behavior under compression.

Figure 1 shows that all the curves exhibit an initial, nearly linear region, indicating elastic behavior. This initial phase extends roughly up to 0.5 mm of displacement. Beyond this point, the curves transition into a nonlinear region, where the force increases more rapidly with increasing displacement. This suggests that the material begins to deform plastically, meaning it retains some permanent deformation after the load is removed.

It’s important to note that the curves for formulations S2 and S4 display a slightly steeper initial slope compared to S1, S3, and S5. This steeper slope suggests a higher stiffness for formulations S2 and S4 in the elastic region. This observation aligns with the findings from the compressive strength measurements, where S2 and S4 indeed exhibited higher compressive strength values compared to the other formulations.

Following this analysis, the compressive strength and compressibility of each formulation were measured.

#### Compressive strength

**Fig. 2:**
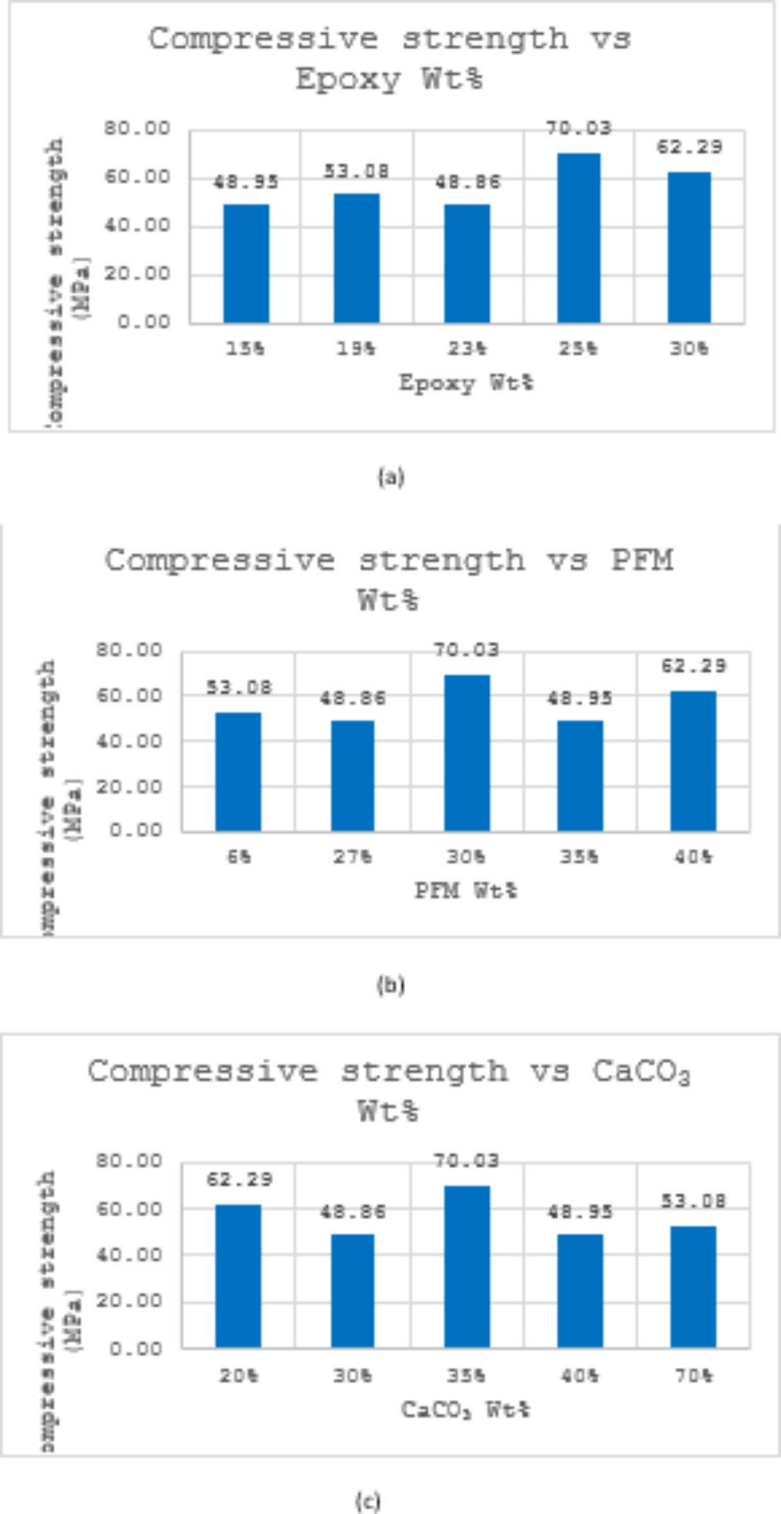
(**a**) Variation of compressive strength with Epoxy Wt%; (**b**) Variation of compressive strength with PFM Wt%; (**c**) Variation of compressive strength with CaCO_3_ Wt%.

Figure 2 presents the correlation between the compressive strength of brake pads manufactured using PFM and the percentage of each ingredient. The data does not reveal any direct relationship between epoxy, PFM, and CaCO_3_ content and compressive strength. S4 has exhibited the highest strength at 70 MPa at 25% epoxy, 30% PFM, and 35% CaCO_3_. Conversely, samples S1 and S3 displayed the lowest compressive strength of 48.9 MPa both samples have almost the same content of PFM and CaCO_3_. It’s crucial to note that additional factors such as PFM composition, manufacturing process, and testing conditions might influence these results. The entire data of compressive strength can be found as Supplementary Table S1 online.

#### Compressibility

**Fig. 3:**
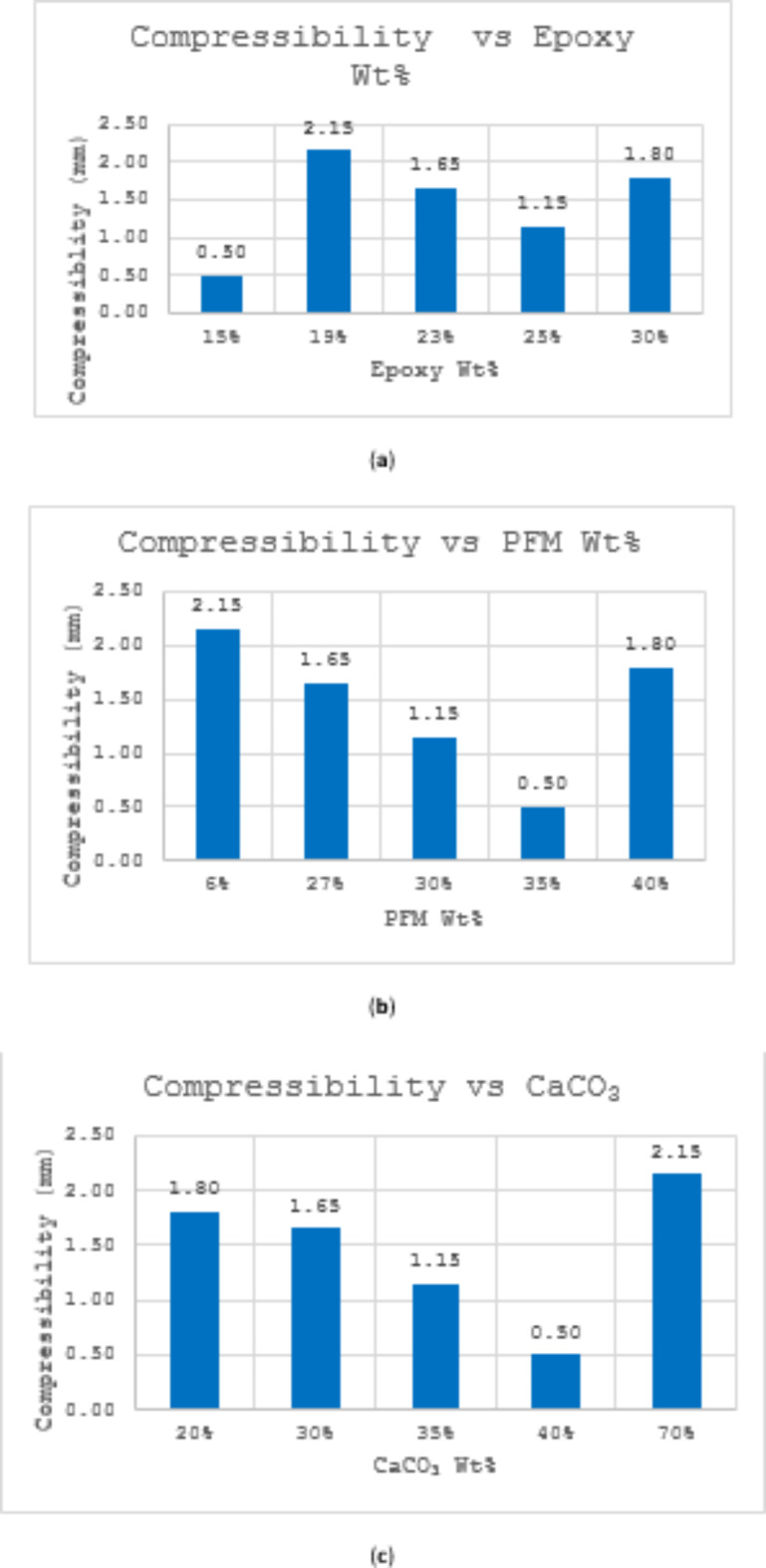
(**a**) Variation of compressibility with Epoxy Wt%; (**b**) Variation of compressibility with PFM Wt%; (**c**) Variation of compressibility with CaCO_3_ Wt%.

Figure 3 shows the variation of compressibility for brake pads produced from PFM along with the weight% of each ingredient. Samples S1 scored 0.5 mm the lowest compressibility value followed by S4 (1.15 mm) while S2 scored the highest compressibility value (2.15 mm).In S1 and S3 both PFM and CaCO_3_ have very close content with difference between them 5%. While S4 has the lowest PFM 6% and the highest CaCO_3_ 70%. It could be observed that as PFM and CaCO_3_ content increase compressibility decreases. Besides there is a decline in results as epoxy increase from 19 to 25% followed by boosting up. The entire data of compressive strength can be found as Supplementary Table S1 online.

#### Hardness

**Fig. 4:**
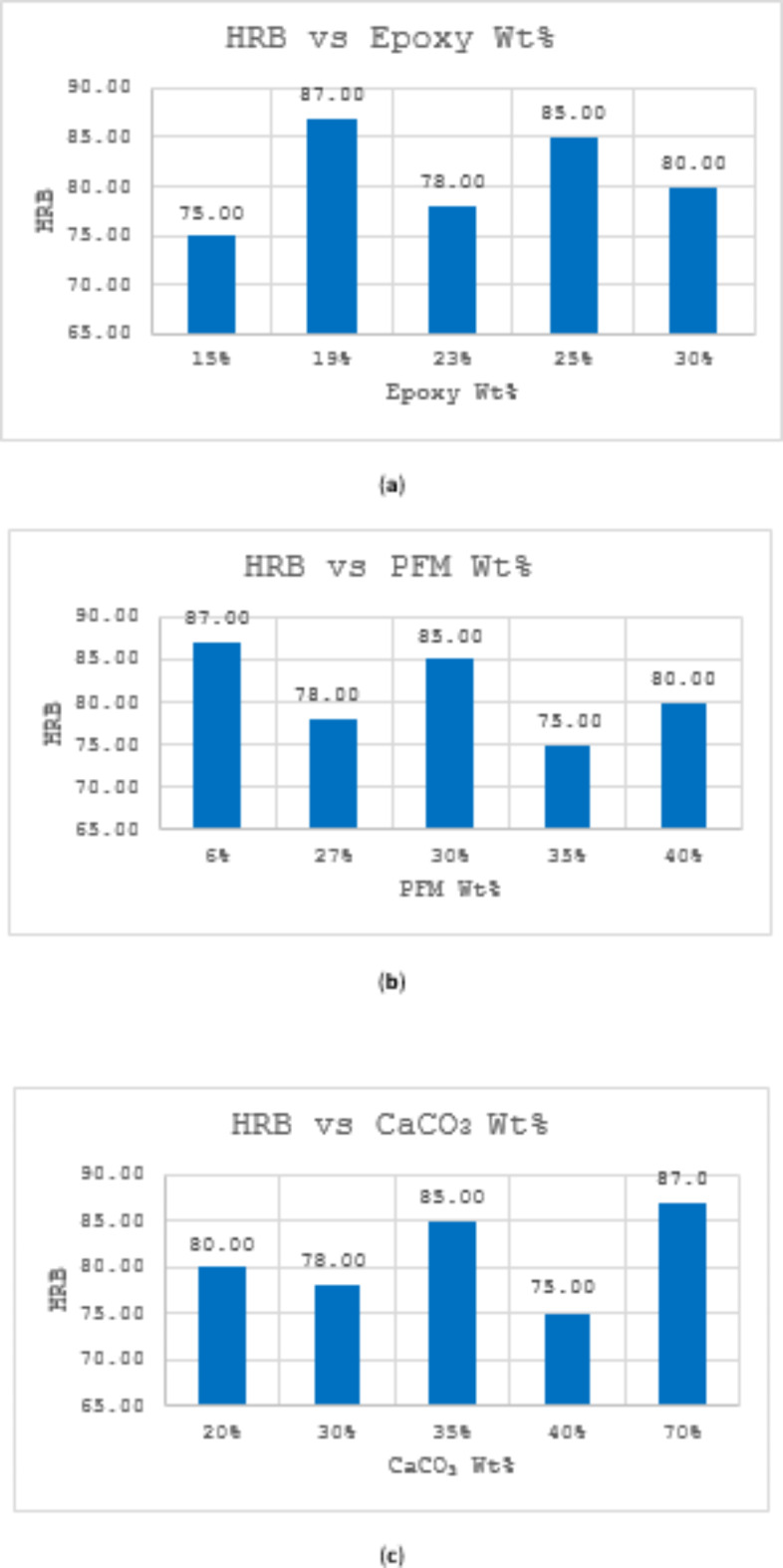
(**a**) Variation of Rockwell hardness B scale with Epoxy Wt%; (**b**) Variation of Rockwell hardness B scale with PFM Wt%; (**c**) Variation of Rockwell hardness B scale with CaCO_3_ Wt%.

Figure 4 presents the Rockwell hardness B values of the produced brake pads, which were determined using a digital hardness tester. Hardness, a crucial property influencing brake pad durability and wear resistance, fluctuated between 75 and 87 HRB across the samples. Sample S2 exhibited the maximum hardness (87 HRB) with the lowest PFM content and the highest CaCO_3_ percentage, while S1 displayed the minimum hardness (75 HRB) at the lowest epoxy content and comparable PFM and CaCO_3_ levels. Notably, no discernible trends were observed between hardness and the weight percentages of individual ingredients, suggesting that the interplay of multiple components might be influencing the hardness values.

### Tribological properties

#### COF

**Fig. 5:**
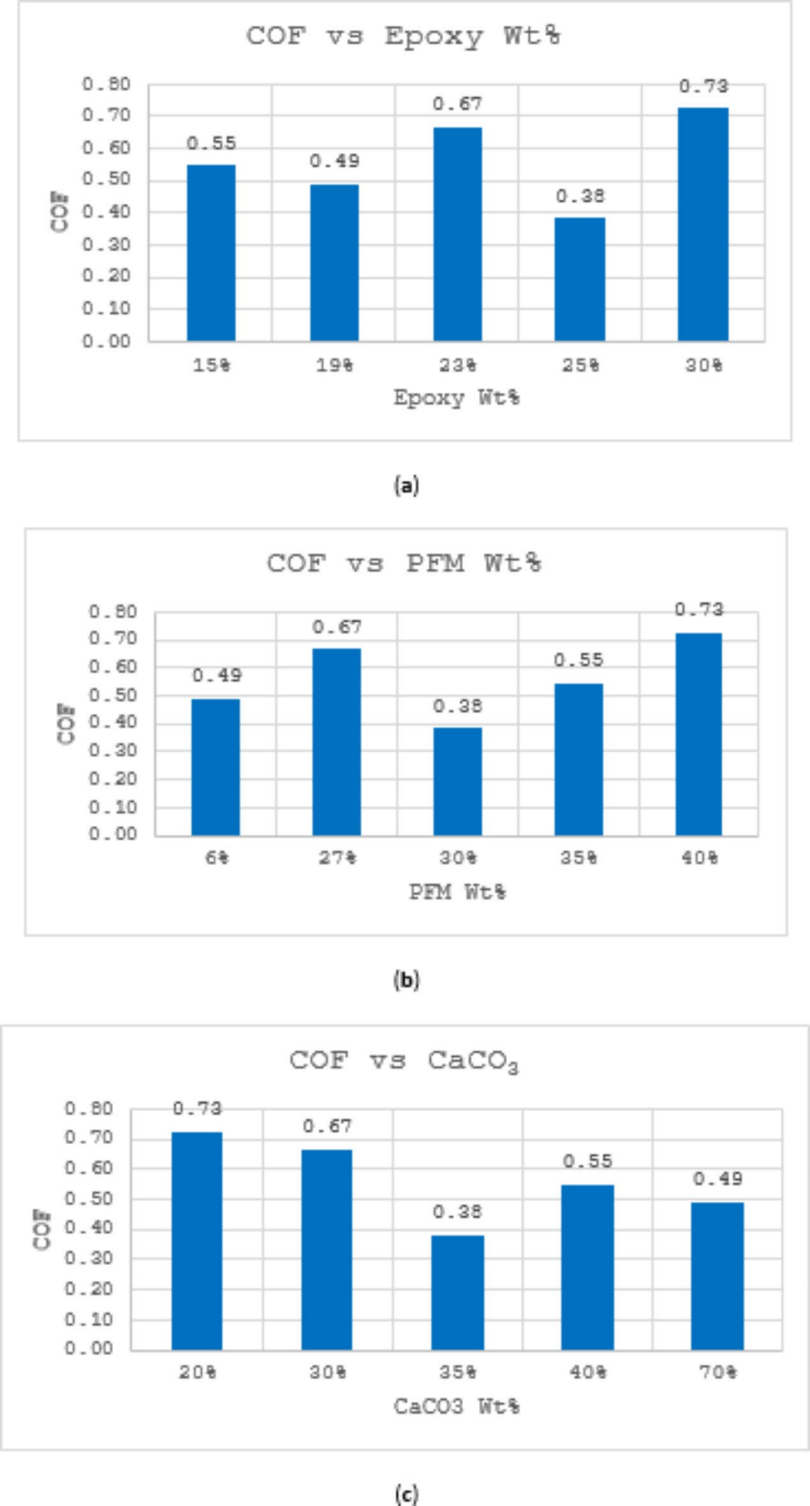
(**a**) Variation of COF with Epoxy Wt%; (**b**) Variation of COF with PFM Wt%; (**c**) Variation of COF with CaCO_3_ Wt%.

Figure 5 presents the coefficient of friction (COF) values for the produced brake pads, ranging from 0.38 to 0.73. Sample S5 exhibited the highest COF at 0.73, while S4 displayed the lowest at 0.38. The lowest COF was observed in the formulation containing 25% epoxy, 30% PFM, and 35% CaCO_3_, whereas the highest COF corresponded to a composition of 20% CaCO_3_ and 40% PFM. A clear inverse relationship exists between CaCO_3_ content and COF, indicating that as CaCO_3_ increases, COF decreases. Conversely, a positive correlation is evident between PFM content and COF, suggesting that higher PFM levels lead to increased COF. These findings highlight the significant influence of CaCO_3_ and PFM on the frictional behavior of the brake pads.

#### Wear rate

**Fig. 6:**
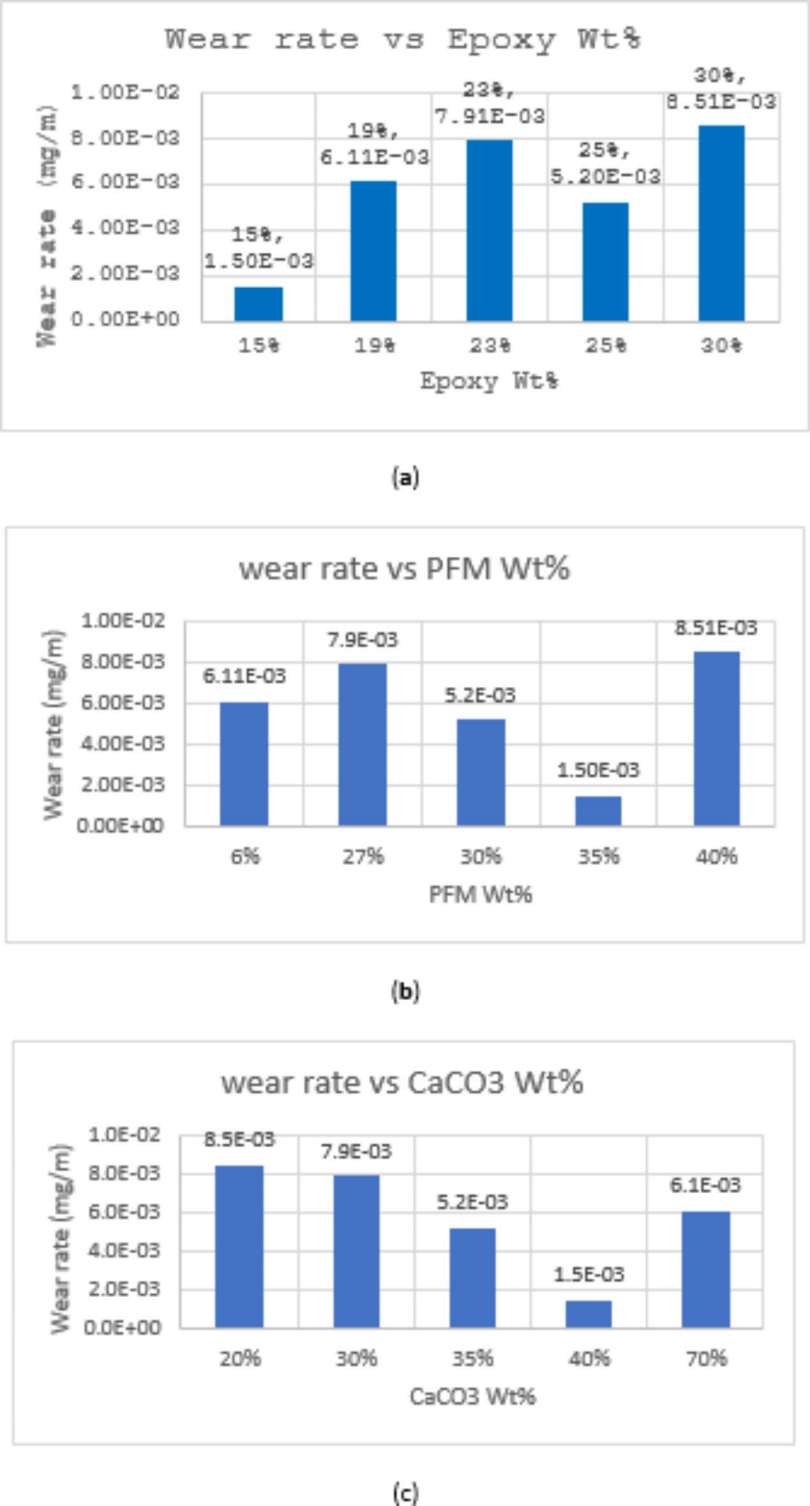
(**a**) Variation of Wear rate with Epoxy Wt%; (**b**) Variation of Wear rate with PFM Wt%; (**c**) Variation of Wear rate with CaCO_3_ Wt%.

Figure 6 illustrates the relationship between wear rate and the composition of the produced brake pads. Among the samples, S1 and S4 exhibited the lowest wear rates, while S5 displayed the highest, followed by S3. A general trend was observed where increasing CaCO_3_ content corresponded to a decrease in wear rate, with a particularly pronounced reduction in S1. Regarding PFM, a rise in wear rate was typically associated with higher PFM percentages, except for S1 and S4, which showed lower wear rates at specific compositions: 15 and 25% epoxy, respectively, combined with 35 and 40% CaCO_3_. These findings suggest that the interplay between PFM, CaCO_3_, and epoxy content significantly impacts the wear behavior of the brake pads.

## Discussion

This study introduces PFM as a sustainable and eco-friendly alternative to traditional brake pad materials. Date palm fibers possess inherent properties that contribute to enhanced mechanical and tribological performance in brake applications. Specifically, their inclusion in brake pads not only addresses environmental concerns associated with asbestos but also offers promising mechanical advantages. This innovative approach underscores our commitment to sustainable engineering solutions in automotive applications. This study examined the influence of material composition on the performance of newly formulated brake pads compared to a commercial asbestos-based pad (Table 3)^51,54^. Key findings include:

### Compressive strength

Epoxy weight% significantly affects compressive strength (Table 4). As epoxy resin serves as the binder that holds the composite materials together, providing cohesion and structural integrity. The proportion of epoxy in the formulation directly impacts the density and internal bonding strength of the brake pad material. Higher epoxy content generally leads to increased cross-linking within the matrix, enhancing the composite’s ability to resist compressive forces. Conversely, lower epoxy content can result in a less densely packed structure, reducing the material’s overall compressive strength. Moreover, the mechanical properties of epoxy resin itself play a crucial role. Epoxy resins are known for their high strength and stiffness, which contribute to the composite’s ability to withstand compressive loads. Variations in epoxy weight% can alter the balance between rigidity and flexibility in the brake pad material, affecting how it responds to compressive stress during braking. Optimal epoxy content ensures that the composite material maintains its structural integrity without becoming too brittle or too flexible. This suggests that optimizing epoxy content is crucial for achieving the necessary structural integrity under braking forces.

New formulations (S1–S4) exhibited lower compressive strength compared to the commercial pad, but achieved significantly reduced wear rates, indicating improved durability (Table 3). This suggests that while epoxy content is crucial for compressive strength, it also plays a significant role in wear resistance. The epoxy resin enhances the bonding between friction materials, which can reduce material loss during braking and improve overall durability. The improved wear rates in the new formulations could be attributed to a more effective distribution of stress and better adhesion between the composite components, leading to less material degradation over time.

### Compressibility

Increasing the weight percentages of PFM and CaCO_3_ influences compressibility values due to several key reasons. Firstly, PFM, being fibrous in nature, can alter the interstitial spaces within the brake pad material matrix. As their percentage increases, these fibers can either compress or interlock differently under pressure, thereby affecting overall pad compressibility. Secondly, calcium carbonate, often used as a filler material, can change the overall density and porosity of the brake pad material. Higher concentrations of CaCO_3_ can lead to increased stiffness or rigidity in the pad, affecting how it compresses when subjected to braking forces. Careful calibration of these components is essential for managing brake pedal feel and responsiveness.

### Hardness

Compared to commercial asbestos-based brake pads, the newly formulated material exhibited higher hardness. Several reasons can be suggested for this influence. Firstly, the inclusion of PFM as a primary component in the new formulations likely contributes to the increased hardness. PFM has distinct mechanical properties, including high tensile strength and rigidity, which can lead to a harder composite material when integrated into the brake pad matrix. The fibrous nature of PFM can create a denser, more interlocked structure, enhancing the overall hardness of the brake pad.

Secondly, the increased concentration of CaCO_3_ in the formulations can also be a contributing factor. CaCO_3_ is a hard, brittle material that, when added in significant quantities, can increase the overall hardness of the composite. The presence of CaCO_3_ can fill voids within the material matrix, reducing its compressibility and increasing its resistance to deformation under load. Additionally, the epoxy resin used as a binder in the new formulations can influence hardness. Epoxy resins are known for their strong adhesive properties and can cure to form a hard, rigid matrix. Depending on the epoxy’s formulation and the curing process, the resulting composite material can exhibit higher hardness compared to traditional asbestos-based pads, which often used phenolic resins with different curing characteristics and mechanical properties. Moreover, as the weight% of epoxy exceeds 25%, there is a decline in hardness. This observation aligns with a documented a reduction in sample hardness as the epoxy content increased from 25 to 30% ^52^.

The higher hardness observed in the newly formulated materials indicates a potential area for further optimization. Adjusting the proportions of PFM, CaCO_3_, and epoxy resin, or exploring alternative materials and binders, may help achieve a more balanced set of properties that closely mimic those of asbestos-based pads. This ongoing optimization is essential to develop safe, high-performing brake pad materials that can serve as viable replacements for asbestos-based products.

### Coefficient of friction (COF) and wear rate (WR)

Increasing PFM and CaCO_3_ weight percentages leads to a decrease in COF and WR (Table 3). These happen as a result of the inclusion of PFM changes the surface interactions of the brake pad material. As PFM content increases, the fiber distribution creates a smoother contact surface, reducing friction. PFM’s fibrous nature also contributes to this lower COF. Moreover, CaCO_3_ acts as a filler that enhances the material’s density and hardness. Higher CaCO_3_ content results in a smoother braking surface and more even load distribution, further lowering the COF and WR. Furthermore, the presence of epoxy resin as a binder plays a crucial role in improving the overall performance of the brake pads. The epoxy resin enhances the bonding between the friction materials, creating a cohesive and stable structure that resists both friction and wear. This strong bonding ensures that the composite materials remain intact under braking stress, extending the brake pad’s lifespan and maintaining consistent frictional properties.

The findings of this study align with previous research on natural-based composites for brake pad applications. For instance, recent studies have demonstrated that incorporating natural fibers such as banana peel fiber resulted in enhanced wear resistance and mechanical properties, similar to the improvements observed with PFM in our experiments. Specifically, a study highlighted the effectiveness of natural fibers in improving the tribological performance of brake pads, which corroborates the significant wear rate reductions observed in formulations S1 and S4 ^38,55^. Additionally, the role of calcium carbonate as a filler in optimizing tribological properties has been extensively reported, where similar trends were observed in our study^56,57^. The synergy between PFM and CaCO_3_ not only enhanced wear resistance but also maintained a consistent coefficient of friction, which is crucial for reliable brake performance. These comparisons underscore the potential of PFM as a sustainable alternative in brake pad manufacturing, confirming its viability alongside other natural-based composites.

Moreover, the increase in wear rate as epoxy weight% increases, aligns with other findings^52^. Despite the new formulation showing a higher COF than commercial asbestos pads, it outperforms previous non-asbestos formulations^34^. This improvement indicates significant progress in developing effective alternatives to asbestos-based brake pads. The synergy between PFM and CaCO_3_ enhances wear resistance and frictional consistency, moving closer to the performance of traditional asbestos pads^34^.

The high COF and, low wear rate explains the low Compressive strength and high hardness. As the formulated brake pads are harder than the commercial which leads to a decline in compressive strength. These advancements highlight the potential of these materials to optimize brake pad performance, offering durable and eco-friendly alternatives that meet high performance standards. Continued research and optimization will be essential for further enhancing these properties and fully replacing traditional brake pad materials.

**Table 3:** Comparison of the result with existing findings.

	Commercial pad	S1	S2	S3	S4	S5
Compressive strength (Mpa)	110	48.95	53.08	48.86	70.03	62.29
Compressibility (mm)	–	0.50	2.15	1.65	1.15	1.80
Hardness (HRB)	54.3	75.00	87.00	78.00	85.00	80.00
Wear rate (mg/mm)	3.8	1.5E-03	6.11E-03	7.9E-03	5.2E-03	8.5E-03
COF	0.3–0.4	0.55	0.49	0.67	0.38	0.73

## Methods

### Preparation of raw materials

**Fig. 7:**
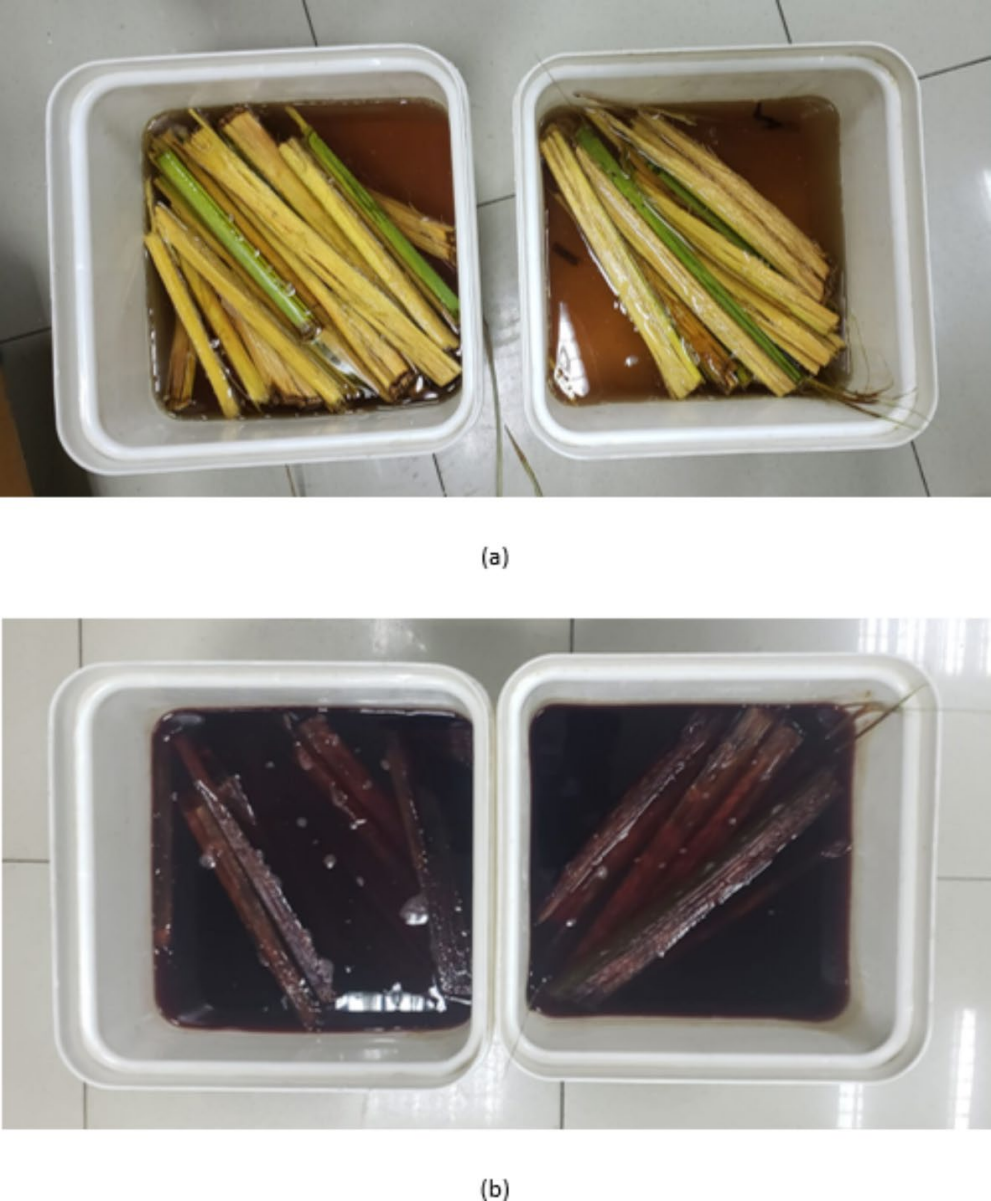
Effect of caustic soda treatment on PFM M: (**a**) Untreated PFM fibers and (**b**) PFM fibers following treatment with caustic soda solution for 24 h.

PD fibers were sourced from the frond midribs (PFM) of Sukari palm dates (Abusir village, Giza, Egypt). They were chemically treated with a 5% NaOH solution for 24 h (Fig. 7). The fibers were then washed with water to remove the caustic soda and sun dried for one week. The dried PFM was grounded into powder form and was thereafter sieved using sieve size ≤ 100 μm aperture.

### Development of the brake pad

The production of brake pad consists of a series of unit operations including mixing, and cold pressing. The sieved PFM was added in varying percentages to aluminum oxide, calcium carbonate, carbon and epoxy resin Table 4. The combinations were separately dry-mixed using a blender in order to achieve a homogeneous state ready for molding.

**Fig. 8:**
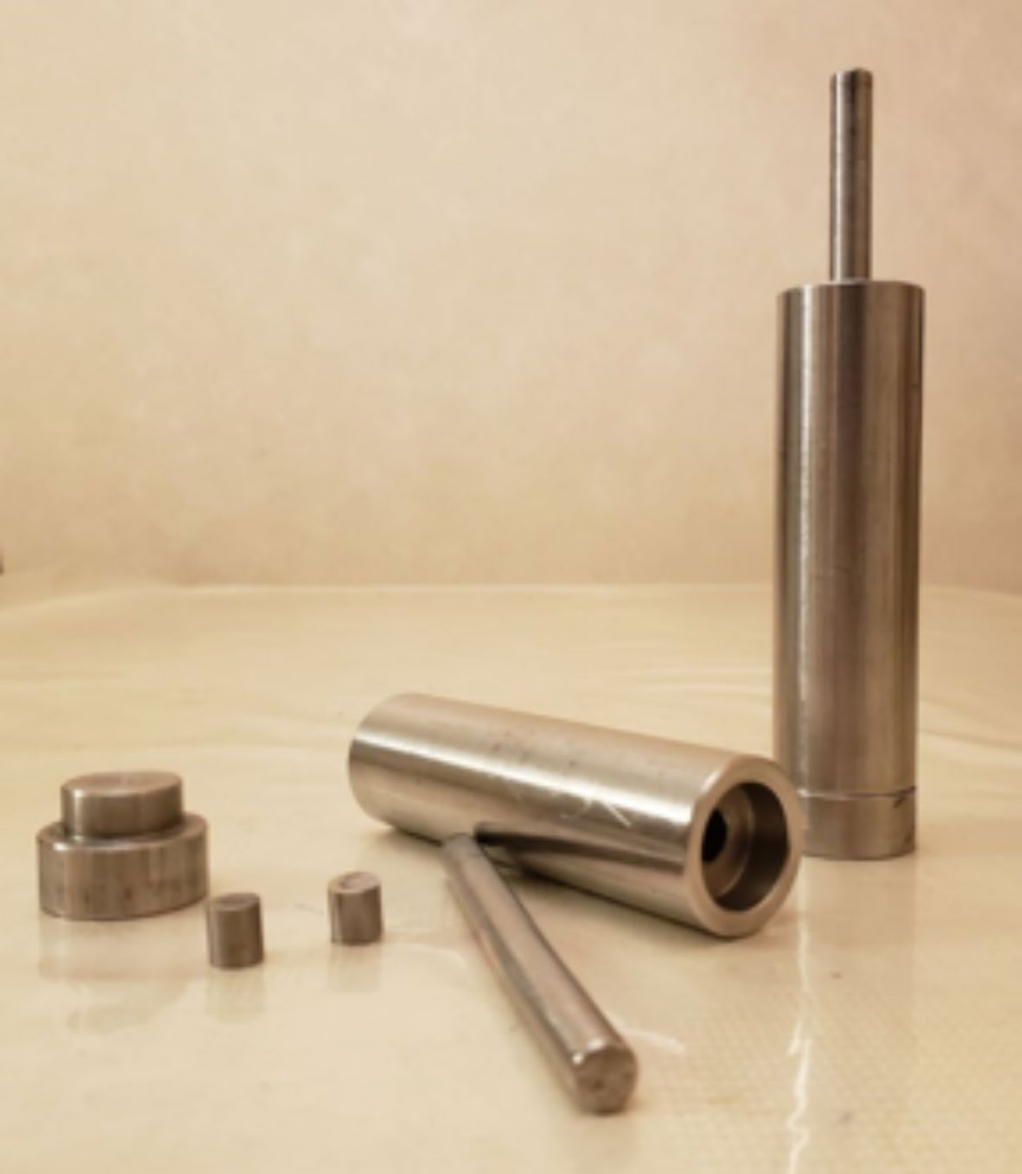
The cold pressing mold components used in pressing samples.

**Fig. 9:**
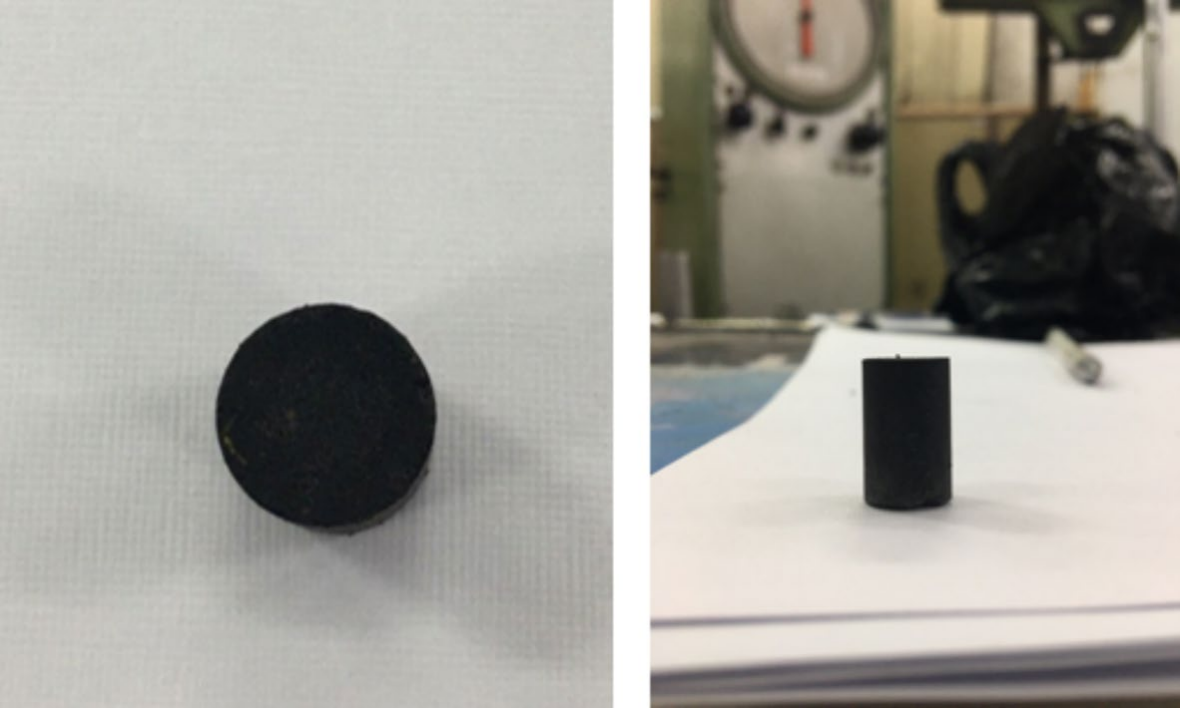
Cylindrical brake pad material samples after cold pressing.

The cold dry pressing mold in Fig. 8 was fabricated from AISI 316 stainless steel to withstand the required pressure. Samples were compressed in the mold at 15 MPa for 2 min using a Wolpert Lestor tension-compression testing machine. The produced samples were finished by polishing them using polisher-grinder with various grinding paper of various sizes to obtain the final products. The produced samples are presented on (Fig. 9).

**Table 4:** weight% of each element in prepared samples.

Ingredients	S1	S2	S3	S4	S5
	Weight%				
Epoxy	15	19	23	25	30
PFM	35	6	27	30	40
Al_2_O_3_	5	0	10	5	5
C	5	5	10	5	5
CaCO_3_	40	70	30	35	20

### Evaluation of the developed brake pads

- Mechanical characterization

Hardness was measured using Rockwell hardness (B scale) SAP180 digital hardness tester. Compressive Strength was measured using a Wolpert Lestor tension-compression testing machine, with compressibility determined using a dial indicator. The video test of compressive strength and compressibility can be found as Supplementary video S2 online.

(b) Tribological characterization

**Fig. 10:**
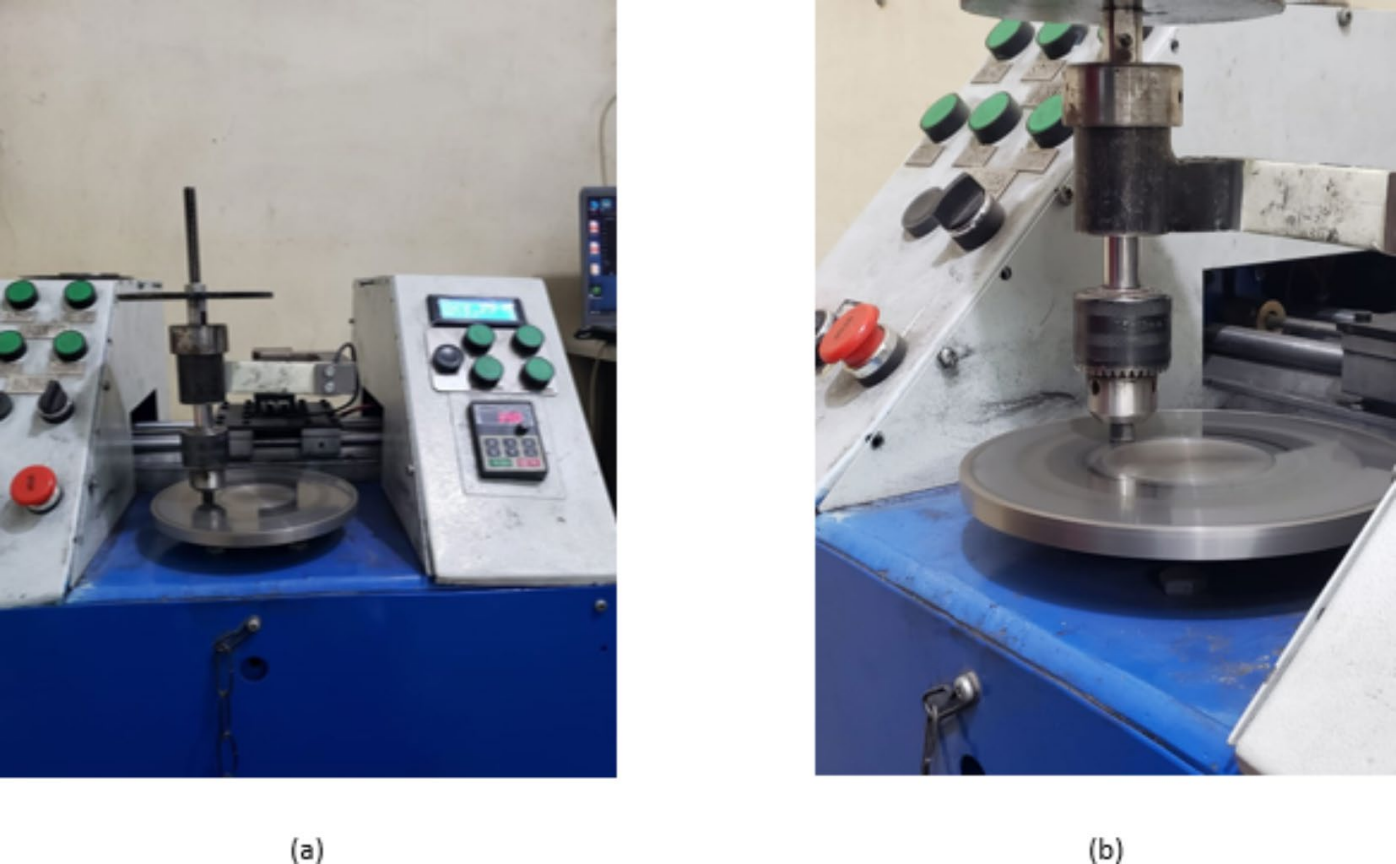
Pin-on-disc machine setup for tribological testing: (**a**) Overview of the machine with disc and specimen holder and (**b**) (a) Close-up of Specimen Mounted in Holder.

Coefficient of friction (COF) and Wear Rate were determined using a pin-on-disc tribometer according to ASTM G-99-95a (Fig. 10) ^58^. Samples were tested against a standard automotive brake disc at 390 rpm, 20 N load, and 1 km sliding distance. Wear rate was calculated using Eq. 1. Samples were weighted before and after the test using electronic scale G&G Model JJ224BC. The video test of testing using Pin-On-Disc machine can be found as Supplementary video S3 online.

## Conclusions

In conclusion, this study explored the potential of date palm fibers, specifically its PFM as a sustainable and eco-friendly alternative for brake pad materials. The research successfully formulated brake pads incorporating PFM and compared their performance to a commercial asbestos-based pad. The analysis revealed several key findings:

- **Improved durability**: While the newly formulated pads exhibited lower compressive strength70.03 MPa compared to the commercial pad 110 MPA, they achieved significantly reduced wear rates up to 1.5 E-03 mg/mm, indicating superior durability. This suggests a trade-off between absolute strength and long-term wear resistance, which can be further optimized.
- **Enhanced hardness**: The inclusion of PFM resulted in increased hardness up to 87 HRB compared to traditional asbestos pads 54.3 HRB. This can be attributed to the inherent strength and rigidity of PFM fibers.
- **Friction and wear optimization**: Increasing the content of PFM and CaCO_3_ led to a decrease in both the COF and wear rate. This is likely due to the smoother contact surface created by PFM fibers and the improved density and load distribution from CaCO_3_.
- **Epoxy resin’s role**: Epoxy resin, the binding agent, plays a crucial role in overall performance. It enhances bonding between friction materials, leading to a cohesive and stable structure that resists wear and tear.
- **Synergistic effects**: The combined effects of PFM, CaCO_3_, and epoxy resin contribute to both lower COF and reduced wear rate. This synergy highlights the potential for these materials to create durable and eco-friendly brake pads.

These findings demonstrate the promising potential of PFM as a sustainable alternative for brake pad production. However, further research and optimization are necessary to achieve performance levels fully comparable to traditional asbestos-based pads. By carefully adjusting the proportions of PFM, CaCO3, and epoxy resin, or exploring alternative materials and binders, researchers can develop a new generation of safe, high-performing, and environmentally friendly brake pad materials.

### Future work

This study has laid a strong foundation for exploring date palm fibers, specifically PFM as a sustainable alternative in brake pad production. To further refine these PFM-based brake pads and ensure their long-term viability, several avenues for future research can be pursued:

- **thermal conductivity analysis**: A deeper understanding of the material’s thermal conductivity is crucial. This will provide insights into heat dissipation during braking, a vital factor for brake performance and pad longevity.
- **Microstructural investigation**: Examining the microstructure of the PFM composites before and after wear and compression testing can reveal valuable information. This analysis can help elucidate wear mechanisms, identify potential failure modes, and guide further material optimization.
- **Expanded mechanical testing**: Measuring additional mechanical properties such as fatigue strength and crack propagation behavior will provide a more comprehensive picture of the material’s performance under various stresses experienced during braking.
- **Alternative binders: phenolic resin exploration**: Investigating the use of phenolic resin as a potential replacement for epoxy resin is worthwhile. Phenolic resins are commonly used in traditional brake pads and offer advantages like high thermal stability. Studying how PFM interacts with phenolic resin can lead to further performance improvements.
- **Chemical analysis of PFM**: A detailed chemical analysis of the PFM fibers is recommended. This analysis can determine the compositional changes in lignin and cellulose content, potentially explaining the observed differences in performance between PFM and other natural fibers like palm kernel fibers (PKS).
- **Spectroscopic analysis**: Employing Infrared (IR) and X-ray Diffraction (XRD) analyses of the PFM fibers, both before and after treatment, can be beneficial. These techniques can offer insights into potential changes in the fiber’s structure as a result of processing or interaction with other composite components. This information can be crucial for optimizing the treatment of PFM for enhanced performance in brake pad applications.

By pursuing these areas of future research, scientists and engineers can further refine PFM-based brake pads, ensuring they meet the high standards required for safe and reliable automotive braking systems while promoting environmental sustainability.

## Electronic supplementary material

Below is the link to the electronic supplementary material.

Supplementary Material 1

Supplementary Material 2

Supplementary Material 3

## Data Availability

Data is provided within the manuscript and supplementary information files.

## References

[1] Tarr, W. R. & Rhee, S. K. Static friction of automotive friction materials. *Wear*** 33**, 373–375 (1975).

[2] Bijwe, J. Composites as friction materials: recent developments in non-asbestos fiber reinforced friction materials—A review. *Polym. Compos.*** 18**, 378–396 (1997).

[3] Callister, W. D. & Rethwisch, D. G. Chapter 16: composites. In: Materials Science and Engineering: An Introduction, (Wiley, 1985).

[4] Satapathy, B. K. & Bijwe, J. Composite friction materials based on organic fibres: sensitivity of friction and wear to operating variables. *Compos. Part. Appl. Sci. Manuf.*** 37**, 1557–1567 (2006).

[5] Ibhadode, A. O. A. & Dagwa, I. M. Development of asbestos-free friction lining material from palm kernel shell. *J. Braz Soc. Mech. Sci. Eng.*** 30**, 166–173 (2008).

[6] Smales, H. Friction materials—Black art or science? *Proc. Inst. Mech. Eng. Part. D J. Automob. Eng.*** 209**, 151–157 (1995).

[7] Spasiano, D. & Pirozzi, F. Treatments of asbestos containing wastes. *J. Environ. Manage.*** 204**, 82–91 (2017). 28863339 10.1016/j.jenvman.2017.08.038

[8] Pini, M. et al. Management of asbestos containing materials: a detailed LCA comparison of different scenarios comprising first time asbestos characterization factor proposal. *Environ. Sci. Technol.*** 55**, 12672–12682 (2021). 34468140 10.1021/acs.est.1c02410 PMC8459455

[9] Tukker, A. Life-cycle assessment and the precautionary principle. *Environ. Sci. Technol.*** 36**, 70A–75A (2002). 10.1021/es022213p 11871577

[10] Guinée, J. B. et al. Life cycle assessment: past, present, and future. Preprint at (2011). 10.1021/es101316v 20812726

[11] Krishna, I. M., Manickam, V., Shah, A. & Davergave, N. Environmental management: science and engineering for industry. Butterworth-Heinemann (2017).

[12] Terazono, A., Moriguchi, Y., Sakai, S. & Takatsuki, H. Environmental impact assessment of sprayed-on asbestos in buildings. *J. Mater. Cycles Waste Manage.*** 2**, 80–88 (2000).

[13] Loss, A., Toniolo, S., Mazzi, A., Manzardo, A. & Scipioni, A. LCA comparison of traditional open cut and pipe bursting systems for relining water pipelines. *Resour. Conserv. Recycl.*** 128**, 458–469 (2018).

[14] Ramesh, M., Deepa, C., Kumar, L. R., Sanjay, M. R. & Siengchin, S. Life-cycle and environmental impact assessments on processing of plant fibres and its bio-composites: a critical review. *J. Ind. Text.*** 51**, 5518S–5542S (2022).

[15] Parfitt, J., Barthel, M. & Macnaughton, S. Food waste within food supply chains: quantification and potential for change to 2050. *Philos. Trans. R Soc. B Biol. Sci.*** 365**, 3065–3081 (2010). 10.1098/rstb.2010.0126 PMC2935112 20713403

[16] Calamari, D., Kimbrough, R. D., Halogenated & Biphenyls Terphenyls, Naphtalenes, Dibenzodioxins And Related Products (1984).

[17] Eriksson, M. & Spångberg, J. Carbon footprint and energy use of food waste management options for fresh fruit and vegetables from supermarkets. *Waste Manage.*** 60**, 786–799 (2017). 10.1016/j.wasman.2017.01.008 28089203

[18] Lam, S. S. et al. Pyrolysis production of fruit peel biochar for potential use in treatment of palm oil mill effluent. *J. Environ. Manage.*** 213**, 400–408 (2018). 29505995 10.1016/j.jenvman.2018.02.092

[19] Sial, T. A. et al. Contrasting effects of banana peels waste and its biochar on greenhouse gas emissions and soil biochemical properties. *Process. Saf. Environ. Prot.*** 122**, 366–377 (2019).

[20] Cruz, J. & Fangueiro, R. Surface modification of natural fibers: a review. *Procedia Eng.*** 155**, 285–288 (2016).

[21] Sanman, S., Manjunath, A., P Prashanth, K., Shadakshari, R. & K Sunil, S. An experimental study on two body abrasive wear behavior of natural fiber reinforced hybrid polymer matrix composites using taguchi analysis. *Mater. Today Proc.*** 72**, 2021–2026 (2023).

[22] Kurien, R. A. et al. A comprehensive review on the mechanical, physical, and thermal properties of abaca fibre for their introduction into structural polymer composites. *Cellulose*** 30**, 8643–8664 (2023).

[23] Kurien, R. A., Selvaraj, D. P. & Koshy, C. P. Worn surface morphological characterization of NaOH-treated chopped abaca fiber reinforced epoxy composites. *J. Bio Tribo Corros.*** 7**, 1–8 (2021).

[24] Mahale, V., Bijwe, J. & Sinha, S. Influence of nano-potassium titanate particles on the performance of NAO brake-pads. *Wear*** 376**, 727–737 (2017).

[25] El-Moayed, M. H., Kühn, J., Lee, S. H., Farag, M. & Mehanny, S. Potential of lignin valorization with emphasis on bioepoxy production. https://arxiv.org/abs/2022

[26] Sanjay, M. R. et al. Characterization and properties of natural fiber polymer composites: a comprehensive review. *J. Clean. Prod.*** 172**, 566–581 (2018).

[27] Rangappa, S. M., Siengchin, S., Parameswaranpillai, J., Jawaid, M. & Ozbakkaloglu, T. Lignocellulosic fiber reinforced composites: Progress, performance, properties, applications, and future perspectives. *Polym. Compos.*** 43**, 645–691 (2022).

[28] Ammar, Z., Ibrahim, H., Adly, M., Sarris, I. & Mehanny, S. Influence of natural fiber content on the frictional material of brake pads—A review. *J. Compos. Sci.*** 7**, 72 (2023).

[29] Teijido, R., Ruiz-Rubio, L., Lanceros-Méndez, S., Zhang, Q. & Vilas-Vilela, J. L. Sustainable bio-based epoxy resins with tunable thermal and mechanic properties and superior anti-corrosion performance. *Polym. (Basel)*** 15**, 4180 (2023). 10.3390/polym15204180 PMC10610945 37896424

[30] Naik, N., Shivamurthy, B., Thimmappa, B. H. S., Guo, Z. & Bhat, R. Bio-based epoxies: mechanical characterization and their applicability in the development of eco-friendly composites. *J. Compos. Sci.*** 6**, 294 (2022).

[31] Agbo, P., Mali, A., Deng, D. & Zhang, L. Bio-oil-based epoxy resins from thermochemical processing of sustainable resources: a short review. *J. Compos. Sci.*** 7**, 374 (2023).

[32] Mgaya, J. et al. Cashew nut shell: a potential bio-resource for the production of bio-sourced chemicals, materials and fuels. *Green. Chem.*** 21**, 1186–1201 (2019).

[33] Yokoyama, Y., Yasui, T., Takeda, A., Ogino, K. & Kanehashi, S. Novel bio-based flexible bisphenol epoxy resin derived from cashew nut shell liquid. *Polym. J.*** 55**, 859–867 (2023).

[34] Ikpambese, K. K., Gundu, D. T. & Tuleun, L. T. Evaluation of palm kernel fibers (PKFs) for production of asbestos-free automotive brake pads. *J. King Saud Univ. Eng. Sci.*** 28**, 110–118 (2016).

[35] Pujari, S. & Srikiran, S. Experimental investigations on wear properties of palm kernel reinforced composites for brake pad applications. *Def. Technol.*** 15**, 295–299 (2019).

[36] Krishnan, G. S., Babu, L. G., Pradhan, R. & Kumar, S. Study on tribological properties of palm kernel fiber for brake pad applications. *Mater. Res. Express*** 7**, 015102 (2019).

[37] Bashir, M., Qayoum, A. & Saleem, S. S. Influence of lignocellulosic banana fiber on the thermal stability of brake pad material. *Mater. Res. Express*** 6**, 115551 (2019).

[38] Bashir, M., Qayoum, A. & Saleem, S. S. Experimental investigation of thermal and tribological characteristics of Brake Pad developed from eco-friendly materials. *J. Bio Tribo Corros.*** 7**, 1–13 (2021).

[39] Al-Shwyeh, H. A. Date palm* (Phoenix dactylifera* L.) fruit as potential antioxidant and antimicrobial agents. *J. Pharm. Bioallied Sci.*** 11**, 1–11 (2019). 30906133 10.4103/jpbs.JPBS_168_18 PMC6394164

[40] Mehanny, S. et al. Extraction and characterization of nanocellulose from three types of palm residues. *J. Mater. Res. Technol.*** 10**, 526–537 (2021).

[41] Al-Karmadi, A. & Okoh, A. I. An overview of date (*Phoenix dactylifera*) fruits as an important global Food Resource. *Foods*. **13**, 1024 (2024). 38611330 10.3390/foods13071024 PMC11011438

[42] Mallaki, M. & Fatehi, R. Design of a biomass power plant for burning date palm waste to cogenerate electricity and distilled water. *Renew. Energy*** 63**, 286–291 (2014).

[43] Pinca-Bretotean, C., Craciun, A. L., Josan, A. & Ardelean, M. Friction and wear characteristic of organic brake pads material. In IOP Conference Series: Materials Science and Engineering, 477, 012009. ( IOP Publishing, 2019).

[44] Craciun, A. L., Pinca-Bretotean, C., Utu, D. & Josan, A. Tribological properties of nonasbestos brake pad material by using coconut fiber. In IOP Conference Series: Materials Science and Engineering, 163, 012014. (IOP Publishing, 2017).

[45] Crăciun, A. L., Heput, T. & Bretotean, C. P. Formulation of materials with natural fiber for brake system components. *Ann. Fac. Eng. Hunedoara*** 14**, 17 (2016).

[46] Maleque, M. A. & Atiqah, A. Development and characterization of coir fibre reinforced composite brake friction materials. *Arab. J. Sci. Eng.*** 38**, 3191–3199 (2013).

[47] Rajan, R., Tyagi, Y. K. & Singh, S. Waste and natural fiber based automotive brake composite materials: influence of slag and coir on tribological performance. *Polym. Compos.*** 43**, 1508–1517 (2022).

[48] Kholil, A., Dwiyati, S. T., Sugiharto, A. & Sugita, I. W. Characteristics composite of wood powder, coconut fiber and green mussel shell for electric motorcycle brake pads. In Journal of Physics: Conference Series, 1402, 055095. (IOP Publishing, 2019).

[49] Kholil, A., Dwiyati, S. T., Wirawan, R. & Elvin, M. Brake Pad Characteristics of Natural Fiber Composites from Coconut Fibre and Wood Powder. In Journal of Physics: Conference Series, 012068 (IOP Publishing, 2021). (2019).

[50] HS, M. F. K. Testing of mechanical characteristics of coconut fiber reinforced for composite brake pads for two-wheeled vehicles. In IOP Conference Series: Materials Science and Engineering, 546, 042018. (IOP Publishing, 2019).

[51] Sutikno, Pramujati, B., Safitri, S. D. & Razitania, A. Characteristics of natural fiber reinforced composite for brake pads material. In AIP Conference Proceedings, 050009 (AIP Publishing LLC, 1983).

[52] Sukrawan, Y., Hamdani, A. & Mardani, S. A. Effect of bamboo weight faction on mechanical properties in non-asbestos composite of motorcycle brake pad. *Mater. Phys. Mech.*** 42**, 367–372 (2019).

[53] Ma, Y. et al. Effects of bamboo fibers on friction performance of friction materials. *J. Thermoplast. Compos. Mater.*** 26**, 845–859 (2013).

[54] Idris, U. D., Aigbodion, V. S., Abubakar, I. J. & Nwoye, C. I. eco-friendly asbestos free brake-pad: using banana peels. *J. King Saud Univ. Eng. Sci.*** 27**, 185–192 (2015).

[55] Manjulaiah, H., Dhanraj, S. & Basavegowda, Y. A novel study on the development of sisal-jute fiber epoxy filler–based composites for brake pad application. Biomass Convers Biorefin 1–13. Preprint at (2023).

[56] Singh, T., Tiwari, A., Patnaik, A., Chauhan, R. & Ali, S. Influence of wollastonite shape and amount on tribo-performance of non-asbestos organic brake friction composites. *Wear*** 386**, 157–164 (2017).

[57] Raghunathan, V. et al. Effective utilization of surface-processed/untreated cardiospermum halicababum agro-waste fiber for automobile brake pads and its tribological performance. *Tribol. Int.*** 197**, 109776 (2024).

[58] ASTM. Standard Test Method for wear testing with Pin on Disk apparatus. ASTM G99-95a (Reapproved 2000).

