# Supplementary material for: Incorporating date palm fibers for sustainable friction composites in vehicle brakes
Source: Sci Rep. 2024 Oct 5;14:23204. doi: 10.1038/s41598-024-73275-1 (PMC11455931; doi:10.1038/s41598-024-73275-1)
Supplement: Supplementary file 3 — Supplementary Material 3 [file 41598_2024_73275_MOESM3_ESM.docx]

| Sample | Avg. diameter (mm) | Force (KN) | displacement µm |
| --- | --- | --- | --- |
| S1 | 10.2 | 3.7 | 15 |
|  |  | 3.8 | 20 |
|  |  | 3.9 | 38 |
|  |  | 4 | 50 |
| S2 | 10.39 | 1 | 45 |
|  |  | 1.8 | 65 |
|  |  | 2.4 | 85 |
|  |  | 3.2 | 105 |
|  |  | 3.9 | 145 |
|  |  | 4.5 | 215 |
| S3 | 10.21 | 0.6 | 100 |
|  |  | 1.6 | 130 |
|  |  | 2.4 | 138 |
|  |  | 3.2 | 150 |
|  |  | 4 | 165 |
| S4 | 10 | 1 | 25 |
|  |  | 1.8 | 45 |
|  |  | 2.4 | 60 |
|  |  | 3 | 70 |
|  |  | 4 | 90 |
|  |  | 5.5 | 115 |
| S5 | 10.31 | 1 | 62 |
|  |  | 1.4 | 68 |
|  |  | 2 | 70 |
|  |  | 3 | 90 |
|  |  | 4 | 110 |
|  |  | 5.2 | 180 |

Table 1: Illustrates samples average diameter, compressibility test data (Force and displacement)
